# Incisional Surgical Site Infection after Elective Open Surgery for Colorectal Cancer

**DOI:** 10.1155/2014/419712

**Published:** 2014-03-27

**Authors:** Kosuke Ishikawa, Takaya Kusumi, Masao Hosokawa, Yasunori Nishida, Sosuke Sumikawa, Hiroshi Furukawa

**Affiliations:** ^1^Department of Plastic and Reconstructive Surgery, Hokkaido University Graduate School of Medicine, Kita 15, Nishi 7, Kita-ku, Sapporo 060-8638, Japan; ^2^Department of Surgery, Keiyukai Sapporo Hospital, Kita 1-1, Hondori 14, Shiroishi-ku, Sapporo 003-0027, Japan

## Abstract

*Background.* The purpose of this study was to clarify the incidence and risk factors for incisional surgical site infections (SSI) in patients undergoing elective open surgery for colorectal cancer. *Methods.* We conducted prospective surveillance of incisional SSI after elective colorectal resections performed by a single surgeon for a 1-year period. Variables associated with infection, as identified in the literature, were collected and statistically analyzed for their association with incisional SSI development. *Results.* A total of 224 patients were identified for evaluation. The mean patient age was 67 years, and 120 (55%) were male. Thirty-three (14.7%) patients were diagnosed with incisional SSI. Multivariate analysis suggested that incisional SSI was independently associated with TNM stages III and IV (odds ratio [OR], 2.4) and intraoperative hypotension (OR, 3.4). *Conclusions.* The incidence of incisional SSI in our cohort was well within values generally reported in the literature. Our data suggest the importance of the maintenance of intraoperative normotension to reduce the development of incisional SSI.

## 1. Introduction

Surgical site infections (SSI) are the most common nosocomial infection in surgical patients, contributing to perioperative morbidity, prolonged postoperative hospital length of stay, and increased hospital costs [1–3]. The colorectal surgery has been associated with the highest risk of SSI [1], predominately because of the heavy bacterial load of the colon and rectum. The incidence of incisional SSI following colorectal surgery has been reported to range from 5% to 26% [4–8]. Although various risk factors for incisional SSI have been reported [1, 4–6], there has been no clear consensus on the risk factors contributing to incisional SSI following colorectal surgery. To clarify the incidence and risk factors for incisional SSI, we conducted prospective surveillance of incisional SSI and analyzed plausible factors in patients undergoing elective open surgery for colorectal cancer, performed by a single surgeon.

## 2. Materials and Methods

### 2.1. Subjects

This prospective observational study was conducted at Keiyukai Sapporo Hospital from January 1, 2009, to December 31, 2009. All patients undergoing elective laparotomy with colorectal resection for cancer by a single board-certified colon and rectal surgeon (Takaya Kusumi) were registered in this cohort. Patients were excluded if the surgical wound was not closed primarily. Also excluded were patients undergoing laparoscopy or simple ostomy creation or closure with associated wedge or segmental resection. The resident surgeon (Kosuke Ishikawa) completed a prospective infection surveillance form for each patient, recording date of identification of incisional SSI. We collected demographic and clinical variables on a standardized form from electronic medical records and operating room records. Approval for this study was obtained from the institutional review board.

The diagnosis of SSI was strictly made based on the definitions stated in the guidelines issued by the Center for Disease Control and Prevention (CDC) [2]. Therefore, wounds with tenderness, swelling, or redness were diagnosed as incisional SSI. The primary outcome measures for this study were the incidence and risk factors for incisional SSI. Secondary outcome measures included number of days to the identification of infection, postoperative length of stay, and microbiology of the infections.

### 2.2. Perioperative Management

Mechanical bowel preparation (MBP) was performed with an oral cathartic agent and an electrolyte solution that did not contain antibiotics in the preoperative period for 1 or 2 days. In case preoperative bowel obstruction was severe, MBP was not performed. Antimicrobial prophylaxis was administered to all patients via intravenous infusion of 1 g of cefmetazole sodium. The exact time for antibiotic infusion was recorded by the anesthesiologist. Antimicrobial prophylaxis was continued postoperatively for up to 48 hours. Anesthesia induction and perioperative care were standardized across all patients. Perioperative normothermia was maintained with standardized use of forced-air heaters.

For surgical site preparation, povidone-iodine scrub was used exclusively. We placed a polyurethane wound protector (the Alexis wound retractor manufactured by Applied Medical) in close contact with a wound margin immediately after making an incision in the abdomen. Our standard anastomotic procedures were functional end-to-end anastomosis [9] using four GIA linear staplers for colectomy, abdominal stapled side-to-end anastomosis [10] using a circular stapler and a TA55 for sigmoid anterior resection, and transanal stapled end-to-end anastomosis [11] using a TA55 or a linear stapler and a circular stapler (double stapling technique [12]) for rectal anterior resection. After completing the anastomosis, approximately 2 L saline lavage of the peritoneal cavity was conducted. After closure of the fascia, we conducted pressure irrigation of the subcutaneous fat tissue with 400 mL of saline solution, using a 20 mL syringe with an intravenous catheter. Abdominal incisions were closed primarily using interrupted absorbable or nonabsorbable sutures for the fascia and nonabsorbable sutures for the skin. A subcutaneous closed-suction drain was placed. Postoperative blood glucose level was evaluated at least once a day until postoperative day 2. All wounds were observed by attending surgeons every day, and, after discharge, the operator observed all wounds in the outpatient setting for at least 30 days postoperatively.

### 2.3. Measures

Patient characteristic data collected included age, gender, body mass index, presence of comorbid medical conditions (diabetes mellitus, cardiovascular disease, and chronic obstructive pulmonary disease), history of prior laparotomy, smoking status, preoperative albumin level, preoperative hemoglobin level, physical status according to the American Society of Anesthesiologists (ASA) score, weight loss of more than 10% body weight within 6 months before the operation, and stage of cancer according to the TNM staging system. Operative variables collected included MBP, timing of prophylactic antibiotic use, type of procedure, type of anastomosis, additional surgical procedures (ostomy creation and multiple organ resection), length of operation, surgical wound classification dichotomized as clean-contaminated (without unusual contamination; class II) or contaminated (gross spillage from the gastrointestinal tract; class III), depth and length of incisional site, intraoperative hypotension (systolic blood pressure <80 mmHg continued more than 5 minutes), estimated blood loss, use of perioperative blood transfusion, and postoperative maximum glucose level. All variables were divided into categorical variables (Tables 1 and 2).

### 2.4. Statistical Analyses

The univariate relation between each independent variable and incisional SSI was evaluated using Pearson's *χ* ^2^ test for categorical variables. The variables with a *P* value <0.2 in the univariate analysis were entered into the multivariate logistic regression model, using a Wald statistic backward stepwise selection. The results of the logistic regression were reported as odds ratios (OR) with 95% confidence intervals (CI). All *P* values were two-tailed, and *P* < 0.05 was considered to indicate statistical significance. All statistical analyses in this study were performed using JMP software (SAS Institute Inc., Cary, NC).

## 3. Results

A total of 224 patients met the inclusion criteria for this study during the 1-year period. The mean patient age was 67 years (interquartile ranges [IQR], 60 to 75), and 120 (55%) were male. Of 224 patients, 33 (14.7%) had a diagnosis of incisional SSI. All were inpatients at diagnosis. The median time to the identification of incisional SSI was 9 days (IQR, 8 to 10). Of 27 infected patients without other postoperative complications, the median postoperative length of stay was 14 days (IQR, 12 to 19) compared with uninfected patients (median, 14 days; IQR, 12 to 18.5; *P* = 0.63).

Tables 1 and 2 summarize the patients' characteristics, perioperative/operative variables, and the incidence of SSI according to the factors. By univariate analysis, patients who developed incisional SSI were more likely to have a higher ASA score and TNM stage. When evaluating the perioperative/operative variables, length of incisional site and intraoperative hypotension were associated with the development of incisional SSI.  Table 3 summarizes the results from the multivariate analysis. For our cohort, factors associated independently with incisional SSI were TNM stage III, TNM stage IV (odds ratio [OR], 2.4), and intraoperative hypotension (OR, 3.4).

Cultures were obtained from 14 of the 33 (42.4%) surgical sites, which resulted in 8 isolates. The bacteria isolated were* Enterococcus spp*. (71.4%), methicillin-resistant* Staphylococcus aureus* (14.3%),* Staphylococcus epidermidis* (7.1%), and* Pseudomonas aeruginosa* (7.1%).

## 4. Discussion

There has been wide discrepancy in the reported incidence of incisional SSI following colorectal surgery, ranging from 5% to 26% [4–8]. Most of this variation is probably due to modification of CDC definitions of SSI and differing personnel performing the assessments for infection. This study attempted to address these potential sources of variation by adopting strict CDC definitions and having prospective infection assessments performed by a single surgeon. We adopted intraoperative antiseptic measures, including wound protector placement [13], syringe pressure irrigation of subcutaneous fat tissue [14], and subcutaneous closed-suction drainage [15]. On this condition, the incidence of incisional SSI was 14.7%, well within values generally reported in the literature (roughly 15%) [6–8, 16, 17].

A further objective of this study was to identify potential risk factors that independently predict development of incisional SSI. Various risk factors for incisional SSI after colorectal surgery have been reported, including a high BMI [4, 16], blood transfusion [5, 18], and ostomy creation [5, 16]. National Nosocomial Infections Surveillance system identified three independent risk factors for SSI: ASA score of 3, 4, or 5, surgical wound classification of contaminated or dirty-infected, and duration of operation lasting more than 3 hours [1]. In our analysis, none of these were identified as risk factors.

By multivariate analysis, we identified TNM stage III and TNM stage IV as a risk factor. This correlation may be due to extent of lymph node dissection. However, a recent study did not identify stage of colorectal cancer as a risk factor for incisional SSI [7]. In our multivariate analysis, we also identified intraoperative hypotension as an operative risk factor. Smith et al. [4] also reported intraoperative hypotension as a risk factor and they theorized the contribution of poor wound tissue perfusion related to hypotension. These data suggest the importance of the maintenance of intraoperative normotension in the reduction of SSI. However, intraoperative hypotension may also be a surrogate marker for other factors that were not identified in the variables studied.

The degree of bacterial contamination is fundamental to the risk of SSI. In the colorectal surgery, we hypothesized that the procedures of anastomosis were associated with contamination of the surgical site, because of the heavy bacterial load of the colon and rectum. There was a trend toward developing incisional SSI if the patient underwent functional end-to-end anastomosis for colectomy and end-to-end anastomosis for rectal anterior resection compared to no anastomosis (i.e., abdominoperineal resection and Hartmann's procedure). However, Konishi et al. [6] reported the higher incidence of incisional SSI in rectal surgery than in colonic surgery. Because surgery for rectal cancer is often associated with ostomy formation, preoperative radiation and total mesorectal excision with anastomosis close to the anal verge, all of which could lead to surgery that lasts longer and has greater bacterial contamination.

The use of prophylactic antimicrobial in colorectal surgery has been proven to reduce the infection rate when compared with no-treatment controls [19]. The Surgical Infection Prevention Project developed 3 performance measures: (1) antimicrobial prophylaxis initiated within 1 hour before incision, (2) prophylactic antimicrobial regimen consistent with published guidelines, and (3) prophylactic antimicrobial discontinued within 24 hours after surgery end time [20]. Weber et al. [21] reported that the prophylactic administration 30 to 59 minutes before incision was more effective than administration during the last half hour. In our cohort, administration of prophylactic antimicrobial was conducted 15 to 50 minutes before skin incision in all cases. There was no significant difference between 15 to 29 and 30 to 50 minutes before incision in the development of incisional SSI. In Japan, 2 or 3 days of prophylaxis with parenteral antibiotics for gastroenterologic surgery was the widely accepted standard. Therefore, intravenous antimicrobial prophylaxis was administered postoperatively for up to 48 hours.

Our median time to the identification of incisional SSI was close to other studies [4, 8, 16]. Unlike other studies [7, 22], we could detect no additional postoperative length of stay related to incisional SSI. However, our postoperative length of stay with uninfected patients was longer than other studies of colorectal surgery [7, 16], which masked the influence of infections on hospital stay. We believe that careful observation of wounds for early identification and treatment is important to minimize the influence of incisional SSI.

The microbiology of the infections in the present study was similar to that reported in other studies [16, 17]. Gram-positive aerobic cocci (*Enterococcus spp*. and* Staphylococcal spp*.) accounted for the majority of the isolates. Cefmetazole sodium was active against most of the bacteria identified in the present study.

Because all operations were performed by a single surgeon in an institute, we could minimize interhospital variations, including observer differences, differences in perioperative management, and different environmental factors. We believe that the present study accurately reflects the incidence of incisional SSI in this cohort. However, a larger study is necessary to provide definitive evidence of risk factors for incisional SSI.

## 5. Conclusions

The incidence of incisional SSI in patients undergoing elective open surgery for colorectal cancer in our cohort was well within values generally reported in other studies. The present study identified a higher TNM stage and intraoperative hypotension as risk factors for incisional SSI following elective colorectal resection. Our data suggest the importance of the maintenance of intraoperative normotension to reduce the development of incisional SSI.

## Figures and Tables

**Table 1:** Patient characteriscics.

Characteristic	Total number	Incisional SSI	
		%	*P* value
Age			0.995
<65	90	14.4	
65–74	74	14.9	
≥75	60	15.0	
Gender			0.209
Male	120	17.5	
Female	104	11.5	
Body mass index (kg/m^2^)			0.381
<25	182	13.7	
≥25	42	19.1	
Comorbidities*			
Diabetes mellitus	36	16.7	0.721
Cardiovascular disease	42	14.3	0.927
Obstructive pulmonary disease	32	9.4	0.356
Prior laparotomy	94	12.8	0.480
Current smoking	52	13.5	0.768
Preoperative albumin (g/dL)			0.846
<3.5	12	16.7	
≥3.5	212	14.6	
Preoperative hemoglobin (g/dL)			0.422
<10	38	10.5	
≥10	186	16.0	
ASA score			0.087
1, 2	186	12.9	
3	38	23.7	
Loss of 10% body weight			0.264
Presence	7	0.0	
Absence	217	15.2	
TNM stage			0.058
I and II	115	10.4	
III and IV	108	19.4	

ASA: American Society of Anesthesiologists.

*Comparison between presence and absence of each medical illness.

**Table 2:** Perioperative/operative variables.

Variable	Total number	Incisional SSI	
		%	*P* value
Mechanical bowel preparation			0.549
Presence	172	14.0	
Absence	52	17.3	
Timing of prophylactic antibiotic use			0.773
15–29 minutes before incision	107	14.0	
30–50 minutes before incision	117	15.4	
Type of procedure			0.688
Right-sided colectomy	38	15.8	
Left-sided colectomy	45	20.0	
Transverse colectomy	13	23.1	
Sigmoid anterior resection	50	12.0	
Rectal anterior resection	54	13.0	
Abdominoperineal resection	14	14.3	
Hartmann's procedure	10	0.0	
Type of anastomosis			0.353
Functional end-to-end	93	19.4	
Side-to-end	66	10.6	
End-to-end	41	14.6	
No anastomosis	24	8.3	
Additional procedure			
Ostomy formation	25	8.0	0.314
Multiple organ resection	34	14.7	0.996
Length of operaion (min)			0.202
<90	121	11.6	
90–180	91	19.8	
≥180	12	8.3	
Wound classification			0.639
II	183	14.2	
III	41	17.1	
Depth of incisional site (cm)			0.269
<3	131	13.0	
≥3	69	18.8	
Length of incisional site (cm)			0.098
<20	120	11.7	
≥20	79	20.3	
Intraoperative hypotension (mmHg)			0.010
SBP < 80	138	19.6	
SBP ≥ 80	86	7.0	
Estimated blood loss (mL)			0.533
<100	116	13.8	
100–400	78	18.0	
≥400	30	10.0	
Perioperative blood transfusion			0.319
Presence	41	9.8	
Absence	183	15.9	
Postoperative glucose (g/dL)			0.384
<200	188	13.8	
≥200	36	19.4	

SBP: systolic blood pressure.

**Table 3:** Multivariate analysis of risk factors for incisional SSI.

Variable	OR	95% CI	*P* value
TNM stages III and IV versus I and II	2.4	(1.1–5.8)	0.042
Intraoperative hypotension SBP < 80 (mmHg)	3.4	(1.3–10.7)	0.019

OR: odds ratio; CI: confidential interval; SBP: systolic blood pressure.
